# Lactylation, a Novel Metabolic Reprogramming Code: Current Status and Prospects

**DOI:** 10.3389/fimmu.2021.688910

**Published:** 2021-06-10

**Authors:** An-Na Chen, Yan Luo, Yu-Han Yang, Jian-Tao Fu, Xiu-Mei Geng, Jun-Ping Shi, Jin Yang

**Affiliations:** Department of Translational Medicine Center, Affiliated Hospital of Hangzhou Normal University, Institute of Hepatology and Metabolic Diseases, Hangzhou Normal University, Hangzhou, China

**Keywords:** lactate, lactylation, macrophage, epigenetic, posttranslational modification

## Abstract

Lactate is an end product of glycolysis. As a critical energy source for mitochondrial respiration, lactate also acts as a precursor of gluconeogenesis and a signaling molecule. We briefly summarize emerging concepts regarding lactate metabolism, such as the lactate shuttle, lactate homeostasis, and lactate-microenvironment interaction. Accumulating evidence indicates that lactate-mediated reprogramming of immune cells and enhancement of cellular plasticity contribute to establishing disease-specific immunity status. However, the mechanisms by which changes in lactate states influence the establishment of diverse functional adaptive states are largely uncharacterized. Posttranslational histone modifications create a code that functions as a key sensor of metabolism and are responsible for transducing metabolic changes into stable gene expression patterns. In this review, we describe the recent advances in a novel lactate-induced histone modification, histone lysine lactylation. These observations support the idea that epigenetic reprogramming-linked lactate input is related to disease state outputs, such as cancer progression and drug resistance.

## Background

Cell behavior is dynamically educated by metabolic flux-mediated adaptation to the microenvironment (1). Lactate, the predominant end product of glycolysis, has been regarded as a waste product. Research over the last 30 years has helped us understand that this metabolite, which has novel biological functions, can be useful as a carbon source for cellular metabolism and simultaneously as a signaling molecule (2).

Decades of research have shown that gene expression pattern alterations are based on chromatin remodeling and posttranslational modifications (PTMs), so-called epigenetic changes, which are often heritable and reversible, allowing fine regulation of cell identity (3). Therefore, diverse types of chromatin modifications, such as methylation, acetylation, phosphorylation, glycosylation, ubiquitination, butyrylation, malonylation, succinylation and glutarylation, result in particular configurations that shape the transcriptional machinery. Furthermore, due to the modulation of epigenetic modifications and gene expression, metabolites including acetyl-CoA, α-ketoglutarate (α-KG), S-adenosylmethionine (SAM), β-hydroxybutyrate and nicotinamide adenine dinucleotide (NAD)^+^ have a crucial role in cellular plasticity (4, 5). Importantly, various diseases are often associated with the dysregulation of epigenetic modifications and metabolic activities (6), and the metabolism-chromatin axes may contain numerous new therapeutic targets (7).

Here, the relationship between lactate metabolism and function (8), as well as the evidence supporting the existence and importance of lactate-derived lactylation, are emphasized. Moreover, the therapeutic aspects of lactylation are discussed.

## Updates Regarding Lactate Physiology

### Advanced Opinion of the Warburg Effect

For energy metabolism, glucose-derived pyruvate enters mitochondria to generate acetyl-CoA and further enters the tricarboxylic acid (TCA) cycle to be used in oxidative phosphorylation (OXPHOS). A total of 36 adenosine triphosphate (ATP) molecules per glucose molecule are produced by this process (9). However, two pyruvate molecules are generated from one glucose molecule, only producing two ATP, in highly glycolytic cells and translating into lactate *via* cytosolic lactate dehydrogenases (LDHs) rather than entering the TCA cycle (10). LDH is mainly expressed in tissues with high glycolysis, such as tumors (9). In 1923, Otto Heinrich Warburg discovered that massive lactate was produced from glucose with special reference to tumor cells (1), a phenomenon called the Warburg effect.

With high glycolysis and low OXPHOS, the Warburg effect confers advantages on proliferating cells such as tumor cells. First, for the metabolism of tumor cells, approximately 85% of the intake glucose is converted into lactate, and in mitochondria, approximately 5% of the pyruvate generated by glycolysis is metabolized *via* OXPHOS, prompting rapid ATP synthesis in the cytoplasm (11). Second, glycolysis produces intermediate metabolites that support the biosynthesis of nucleotides, amino acids and lipids, even with the fluctuation of oxygen supply (12). Third, due to decreased reactive oxygen species (ROS) production, the Warburg effect prevents cells from experiencing oxidative stress. In addition, in the conversion of pyruvate to lactate, because of NAD^+^ regeneration, the maintenance of glycolysis is dependent on preservation of the NAD^+^/NADH ratio (13, 14). Finally, enhanced glycolysis leads to improved lactate production, inducing microenvironmental acidification (12, 15, 16).

Adding another layer of complexity to metabolic heterogeneity, in the reverse Warburg effect, stromal cell-derived lactate is transferred to tumor cells, allowing them to generate ATP and thus promoting tumor survival (17, 18). Overall, regarding significant characteristics of cancer, a phenotype of high glucose ingestion and lactate release is still critical (19).

### Lactate Turnover

Lactate is rapidly exchanged throughout the body (20). The lactate concentration can transitorily increase to 15 mM during exercise in humans, while it is usually 1-3 mM at rest. During embryonic development, blastocysts consume approximately 50 to 320 pmol of glucose/embryo/h, and 90% of the consumed glucose is converted to lactate (10). Lactate has the highest circulatory turnover of all metabolites and exceeds that of glucose in mice by 1.1-fold during the fed state and 2.5-fold during the fasted state, as demonstrated by ^13^C-labeled nutrients with intravenous infusions (21). An acute inflammatory response or inadequate perfusion is often known as an underlying pathogenic condition in local lactate accumulation. In arthritis, lactate acts as an amplifier of inflammation inducing modulation of immune cell functions in various ways and production of pro-inflammatory cytokines (22–25). During ischemia, endothelial-derived lactate drive M2-like macrophage polarization to promote muscle regeneration (26).

Indeed, lactate is the most significant metabolic hallmark of tumor cells with high glycolytic activity, even reaching concentrations of approximately 30-40 mM (9). Lactate is reused by different cell populations in the tumor microenvironment (TME), a phenomenon called metabolic symbiosis (27). For instance, normoxic tumor cells in nearby vessels can take up lactate derived from hypoxic and/or glycolytic tumor cells. The conversion of lactate back to pyruvate fuels OXPHOS in these tumor cells, allowing glucose to diffuse towards the more hypoxic cells. This lactate-based symbiotic relationship supports the survival of both hypoxic and normoxic tumor cells under acidic conditions (9, 28).

### The Lactate Shuttle

Lactate function depends on specific receptors, primarily monocarboxylic acid transporters (MCTs), G protein-coupled receptors (*e.g.*, GPR81) (10), and hydroxycarboxylic acid receptor 1 (HCA), present in the brain (29). Mechanical restriction is involved in glucose transport, which is a critical rate-limiting step for glucose uptake in metabolism; conversely, MCTs are almost ubiquitously expressed with free access to lactate for all cells within the body. Changing lactate is accompanied by changing MCT1 protein level. MCT1 has been found predominantly in oxidative muscle (red) fibers, consuming lactate produced by neighboring white muscle fibers for further oxidation (30). And endothelial lactate shuttles to macrophages in an MCT1-dependent manner after muscle ischemia (26). MCT4 is primarily expressed in highly glycolytic cells, such as white muscle fibers, and is upregulated directly *via* hypoxia-inducible factor 1α (HIF-1α) in response to hypoxic conditions to facilitate exportation of lactate (31). Via endothelial GPR81 signaling, bone marrow-derived inflammatory neutrophils promote the release of lactate (32).

As described in the lactate shuttle hypothesis, the linkage with lactate, as the vehicle of exchange between glycolytic and oxidative metabolism, is continuously ongoing among producers and consumers in strict aerobic conditions and can surpass compartmental barriers (33). The shuttle constitutes an intimate connection with lactate symbiosis or the reverse Warburg effect. For instance, lactate produced in peripheral tissues (muscle) is reutilized for glucose formation (gluconeogenesis) in liver, completing the Cori cycle (34). In brain, neurons can metabolize lactate originating from astrocytes, while the glutamate released from neurons is consecutively taken up by astrocytes. As a consequence, glycolysis is in turn stimulated in astrocytes and then lactate moves outward through MCT4 into the extracellular space, driving lactate influx into the surrounding neurons *via* MCT2 (35). Considering the lactate shuttle theory, some clinicians currently understand lactatemia as a biomarker of strain but not stress (33). Lactate might be a promising therapy for patients with acute organ injury. In mice models of hepatitis and pancreatitis, lactate reduces liver and pancreatic inflammatory injury *via* GPR81-mediated suppression of innate immunity (36). Besides, lactate also is being considered as a therapy for dengue fever (37) and sepsis (38).

### Lactic Acid Homeostasis

With the detection of circulating metabolic flow in mice, lactate is considered a major source carbon for the TCA (21), and pyruvate in tissue is converted to circulating lactate quickly. An emerging theory was recently proposed (20): glucose is a specific fuel, and lactate is a universal fuel. The utilization of lactate as the major source of cyclic carbohydrate energy profitably allows glucose to be reserved for use by important systems specifically involved in brain and immune functions.

Next, except the brain, glucose is responsible for TCA metabolism indirectly *via* circulating lactate in all tissues during a fasted condition. In a fasted genetically engineered mouse model of lung and pancreatic tumors, circulating lactate contributes more to the production of TCA cycle intermediates than glucose. Therefore, lactate is a primary circulating TCA substrate (21). In addition, lactate production in adipocytes occurred independent of glucose metabolism. Primary mammalian adipocytes are treated with insulin, which can increase glucose uptake and transformation of lactate, and compared to other metabolites of glucose, conversion to lactate exhibits a stronger response to insulin (39). Furthermore, the import of massive lactate *via* MCT1 takes place in beige adipocytes even if glucose is unlimited. Additionally, the brain endothelium maintains lactate homeostasis and improves adult hippocampal neurogenesis and cognitive functions (40). Thus, lactate homeostasis (41) highlights that lactate plays an important role in fine-tuned cellular metabolism based on extracellular metabolic status and reinforces the function of lactate metabolism for the regulation of energy homeostasis (42).

The function of lactate is dependent on circulating concentration controlled strictly in organism. Barrier-free lactate shuttle underlies the majority of circulating lactate turnover. With the high turnover, lactate is typically well mixed between tissues and circulation that achieve the state of lactate sharing to maintain homeostasis.

## Lactate: a Metabolic Driver in the Microenvironment

Accumulation of lactate in the tissue microenvironment is a feature of both inflammatory diseases and cancer. Lactate has been rediscovered as one of the most evolutionarily ancient signaling molecules and works as a regulator of metabolism, immune reactions, and intercellular communication (43).

### Lactate in the Tumor Microenvironment

The TME concept was proposed due to consideration of the impact on factors other than tumor cells. Notably, lactate homeostasis is seriously disrupted in the TME. The lactagenesis hypothesis in cancer suggests the following orchestrated steps: 1) Increased glucose uptake is determined by elevated expression as well as translocation of glucose transporters. 2) The expression and activity of glycolytic enzymes, especially LDHA, are induced by HIF-1α, c-MYC and p53 dysregulation. 3) Mitochondrial function is decreased primarily by dysregulated p53 expression. 4) Lactate generation, accumulation and secretion are increased because of the mass effect of accelerated glycolysis (44). 5) The physiological level of the intracellular pH in tumor cells is controlled by lactate outflux with H^+^ cotransports *via* upregulation of MCTs, driving the formation of an acidic tumor microenvironment (10).

**Figure 1:**
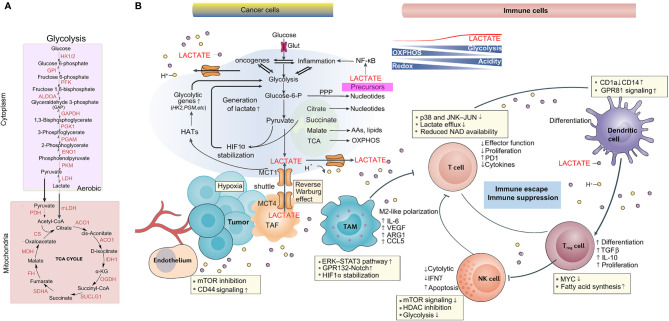
Lactate metabolism is implicated in the tumor microenvironment. **(A)** The main biochemical players in lactate generation: the glycolytic pathway and OXPHOS. **(B)** Role of lactate in the TME, including tumor, stromal, immune cells, blood vessels and cellular metabolites such as lactate. As a consequence of the Warburg effect and the lactate shuttle, lactate accumulation induces acidosis, angiogenesis, immunosuppression, and tumor cell proliferation and survival.

Making space for the expansion of tumor growth and extracellular matrix (ECM) degradation, local tissue invasion, sustained angiogenesis, and migration are required (45).. For instance, lactate in the TME ameliorates conjugation with ECM components and subsequent tumor cell migration by adjusting the binding functions of integrins on tumor cells (46). The increased length and density of tumor cell invadopodia induce the motility and invasiveness of tumor cells on account of the decreased extracellular pH (47). As an angiogenesis promoter, lactate participates in angiogenesis through HIF-1α stabilization to increase vascular endothelial growth factor (VEGF) expression (48–50) (**Figure 1**).

### Lactate in the Inflammatory Microenvironment

Lactate accumulation and acidification are also evolutionarily preserved phenomena that occur during local inflammation. Under the restriction of T cells on sites of inflammation, the high lactate and low pH in inflamed tissues with hypoxia may be highly advantageous in the acute setting. However, suppressing the cytolytic function of CD8^+^ T cells or modulating the CD4^+^ T cell phenotype towards Th17 cells during chronic inflammation might be harmful (51).

In rheumatoid arthritis patients, MCT1-driven lactate uptake into CD4^+^ T cells results in increased interleukin 17 (IL17) production *via* nuclear pyruvate kinase M (PKM2)/signal transducer and activator of transcription 3 (STAT3) signaling. The measurement of synovial lactate is a rapid diagnostic approach used to verify patients with septic arthritis (52).

### Effects of Lactate on Immune Cells

Generally, research suggests that lactate has immune depressive effects on most immune cells of various types in the TME. In brief, tumor-derived lactate was found to promote the development of myeloid-derived suppressor cells (MDSCs) (53) and to allow immunosuppressive cell polarization in dendritic cells (54). In addition, the proliferation, function and movement of cytotoxic T cells (CTLs) are thought to be suppressed by lactate (55), causing injured recruitment of CTLs into the TME. Moreover, lactate decreased IFN-γ production and the cytotoxic activity of natural killer (NK) cells. In contrast, NK cell apoptosis is caused by lactate-driven acidification of the TME (56). The microenvironment with high lactate does not favor the proliferation, survival, and effector function of natural killer T (NKT) cells (57).

### Effects of Lactate on Macrophages

Macrophages, a heterogeneous cell population, act as scavengers, modulate the immune response and maintain tissue homeostasis (58). Macrophage plasticity is induced partially through the maintenance of phenotype stability with regulation of epigenetic dynamics in the settings of inflammation, autoimmunity and cancer (59). Typically, activated macrophages are classified into two phenotypes based on the polarization state: M1 macrophages tend to be a proinflammatory phenotype and obtain energy through glycolysis, and M2 macrophages are predominantly present in immune regulation and tissue repair (60).

During the initiation of tumor development, tumor-associated macrophages (TAMs) often exhibit proinflammatory and antitumor activities (60). However, with tumor progression, macrophage polarization normally skews to the M2 phenotype (60). Tumor-derived lactate induces the polarization of TAMs towards an anti-inflammatory, proangiogenic phenotype with the expression of arginase 1 (Arg1) and VEGF (61) *via* the stabilization of HIF-1α and a reduction in the level of the cofactor NAD^+^, which inhibits pADP-R synthesis (9). Next, uptake of tumor-derived lactate is caused by macrophages again due to MCTs. These MCTs are crucial for tumor cell-macrophage lactate shuttling as well as for macrophage recruitment and reeducation towards a proangiogenic, tumor-promoting phenotype (**Figure 1**).

## Lactylation: A Novel Epimetabolic Code

### Glycolysis and Epigenetic Regulation

Cellular plasticity is derived from the ability to respond and adapt to the environment (62), as demonstrated by macrophage polarization. The activation of macrophages to an inflammatory phenotype is driven by enhanced glycolysis, but energy metabolism is relevant to OXPHOS and β-oxidation in homeostatic macrophages (63). Trained immunity, as a new phenomenon, has been proposed according to research within the last decade and is thought to be a perennial function of innate immune cells in the reprogramming process (64). Trained immunity promotes epigenetic glycolytic gene activation, motivating stimulation of aerobic glycolysis in myeloid cells (65).

Since the regulatory mechanism by which epigenetic control of transcription is strict, metabolic pathways are obviously modulated. For example, blocking glycolysis increases H3K4me3 and decreases H3K9me3 in the IL6 and TNFA gene promoters, thereby inhibiting the training of human monocytes (66). Next, the activity of epigenetic enzymes can be modified by intermediate products of energy metabolism. Furthermore, the activity of two enzyme families controlling epigenetic modifications depends on the ratio of α-KG and succinate generated from the TCA cycle during macrophage activation (67). Additionally, DNA demethylation is associated with the Jumonji domain-containing (JMJ) family of lysine demethylases and the ten-eleven translocation (TET) family of methylcytosine hydroxylases (68).

Regarding histone acetylation (69), it has been reported that alterations in the glycolysis rate advantageously weaken histone acetylation *via* the regulation of acetyl-CoA, which is not only a metabolite but also a substrate of acetyltransferases (70). Additionally, some lysine acylation modifications, including histone butyrylation, propionylation, and 2-hydroxyisobutyrylation, cope with alterations in the glycolysis rate (70). Other research has demonstrated that acetyl-CoA synthesis is regulated through the modulation of enzymes, including acetyl-CoA synthetase 2 and ATP citrate lyase, which contribute to controlling histone acetylation (71).

Lactate plays a significant role in epigenomic reprogramming, which has been shown directly in pancreatic ductal adenocarcinoma (72). Metabolite tracers showed that tumor-derived lactate promotes an increase in α-KG production by mesenchymal stem cells (MSCs). In contrast, demethylase TET activation is mediated by α-KG, resulting in decreased cytosine methylation and increased hydroxymethylation during *de novo* differentiation of MSCs to cancer-associated fibroblasts (CAFs).

Lipopolysaccharide (LPS) is often used to evoke a proinflammatory response in macrophages. LPS tolerance is altered substantially under the modulation of epigenetic mechanisms at the molecular level. It is unclear whether the molecular mechanisms are involved in tolerance drivers; however, recruitment of the nuclear receptor corepressor (NCOR)-histone deacetylase 3 (HDAC3)-p50 suppressive compound to targeted genes in bone marrow-derived macrophages (BMDMs) is related to NF-κB (73). In fact, the response of BMDMs to LPS *in vitro* requires HDAC3, and IL-4 and transforming growth factor (TGF)-β signaling are inhibited in the BMDMs of HDAC3 knockout mice (74).

The results from both *in vitro* and *in vivo* assays have shown that lactate is an endogenous HDAC inhibitor that induces HDAC-dependent gene deregulation at concentrations that arise in nature (75). The addition of lactate decreases the nuclear content and activity of HDAC (76) and the methylation and compaction of chromatin (75).

Collectively, the studies conclude that transcription acts on a permissive chromatin conformation due to augmented lactate, which provided evidence for the earlier discovery by Hashimoto and Brooks that lactate added to L6 cells increased DNA binding (77). Thus, epigenetic modification can constitute an integrated joint during lactate-induced polarized cells under either physiological or disease conditions (78).

### Discovery of Lactylation

In 2019, a novel histone acylation code known as lactylation was proposed (79). All four canonical histones of lysine lactylation in HeLa cells and BMDMs were identified by mass spectrometry. As an indication that exogenous and endogenous lactate contributes directly to lysine lactylation, glycolysis inhibitors with exhausted lactate decrease lysine lactylation, while mitochondrial inhibitors or hypoxia with increased lactate production amplify lysine lactylation. Moreover, the ^13^C-glucose tracer proved that the incorporation of glucose-derived carbon into lysine lactylation (Kla) instead of lysine acetylation (Kac) was reduced by lactate inhibition. In addition, when LDH was genetically deleted, lysine lactylation was abolished (79).

Collectively, the study found increased histone lactylation in human cell lines with exposure to agents or conditions of augmented cellular lactate levels, such as (1) glucose supplementation; (2) the inhibitor rotenone, which promotes glycolysis; (3) hypoxia; and (4) M1-polarized macrophages. On the basis of these studies, a novel histone modification with special modulation of lactate is established and suggests that lysine lactylation is regulated by alterations in glucose metabolic dynamics of glucose and lactate levels.

### Lactylation in Inflammation

During M1 macrophage polarization, histone lactylation increases in a pattern closely correlated with time (79), which is a characteristic of M1 macrophages rather than M2 macrophages. This finding emphasizes that active glycolysis is important to the modulation of histone lactylation.

B cell adapter for PI3K (BCAP) functions as the upstream regulator of Toll-like receptors (TLRs) and is required for optimal glycolysis after microbial stimulation (80). In dextran sulfate sodium (DSS)-induced inflammatory colitis, research showed that wild-type (WT) BMDMs produced more lactate than BCAP^-/-^ BMDMs in response to TLR binding (81). The addition of exogenous lactate rescued this defect of BCAP-deficient BMDMs to enhance histone lactylation and undergo a transition to a reparative phenotype, as demonstrated by the upregulation of Arg1 expression by 12 h and Klf4 by 48 h (80).

Indeed, to evaluate the functional purpose of lactylation, RNA sequencing was conducted, and there was notable enrichment of genes enhanced by lysine lactylation in wound healing, especially homeostasis genes, which are essential to maintain biological homeostasis and are related to M2-like macrophages. For instance, in M1 macrophages treated with exogenous lactate, lysine lactylation enrichment at the Arg1 promoter and increased gene expression were observed. Analogously, histone lactylation and Arg1 expression decreased because of the absence of LDHA in M1 macrophages.

These data revealed that the produced lactate is necessary for proper histone lactylation that induces stimulation of gene expression, resulting in the promotion of an M2-like phenotype at later stages of M1 macrophage polarization, eventually maintaining homeostasis.

### Lactylation in Fibrosis

Convincing evidence has indicated that upregulation of glycolysis in lung fibroblasts, smooth muscle cells and endothelial cells contributes to the progression of lung fibrosis (82, 83). In the lungs of pulmonary fibrosis mice treated with bleomycin and humans with idiopathic pulmonary fibrosis, histone lactylation was increased. Histone lactylation at the promoter-proximal regions of reparative genes such as Arg1, platelet-derived growth factor (Pdgf), thrombospondin 1 (Thbs1), and Vegf was significantly increased in lactate-treated macrophages (84).

### Lactylation in Differentiation

In somatic cells, a metabolic shift from OXPHOS to a state that depends on glycolytic flux during induced pluripotent stem cell (iPSC) processes has been observed (85, 86).

Gli-like transcription factor 1 (Glis1), a member of the Krüppel-like zinc finger transcription factor subfamily, enables senescent cells to convert to pluripotent cells in cellular reprogramming by multilevel epigenetic and metabolic remodeling. At earlier stages, Glis1 shuts off somatic genes such as SET binding protein 1 (Setbp1) and Thy-1 cell surface antigen (Thy1) while turning on glycolysis genes. Owing to enhanced glycolytic flux, the levels of acetyl-CoA and lactate in cells increase, thus promoting acetylation (i.e., H3K27Ac) and lactylation (i.e., H3K18 la) at the loci of pluripotency genes, mainly the Yamanaka factors POU class 5 homeobox 1 (Oct4), SRY-box transcription factor 2 (Sox2), Krüppel-like factor 4 (Klf4) and MYC proto-oncogene (c-Myc), to facilitate cellular reprogramming (87). Additionally, Glis1 is involved in oncogenic processes, including the control of cell proliferation, differentiation, self-renewal, and epithelial-mesenchymal transition (88).

### Lactylation in Cancer

Studies have shown that in BMDMs, treatment with lactate derived from tumor cells helps to drive TAM polarization to an M2-like phenotype *via* the expression of M2 genes, such as Arg1 (61).. Furthermore, using mouse cancer models from B16F10 melanoma and LLC1 lung tumors, one group found that Arg1 expression positively correlated with histone Kla levels, but not Kac levels, in isolated TAMs (79).

### Lactylation of Nonhistone Proteins

Beyond histone protein modifications, a global lysine lactylome analysis was recently performed by LC-MS/MS in *Botrytis cinerea*, a devastating necrotizing fungal pathogen. There were 273 identified Kla sites from 166 proteins, and most proteins with lactylation had wide distribution, including the nucleus (36%), mitochondria (27%), and cytoplasm (25%) (89).

For instance, MAPK was identified to be lactylated at K60. Citrate synthase is a rate-limiting enzyme in the TCA cycle and was found to be lactylated at K401. At multiple sites of two eukaryotic translation initiation factor 5A (eIF5a), proteins were modified by lactate (89). The protein interaction network demonstrated that lactylation could regulate interactions between proteins, highlighting the extensiveness of lactylation within cells.

## Epigenetic Decisions: Lactate and Acetyl-CoA

One caveat in interpreting the connections between lactate metabolism and epigenetic events is that metabolic pathways are interconnected and coordinately regulated (90).

### Coordinated Decisions

Studies in stem cells have identified a role for acetyl-CoA in maintaining the pluripotency program: it was shown that both H3K27Ac and H3K9Ac were enhanced by acetate and that these acetylation modifications were generally diminished during differentiation (4). Mechanistically, glycolysis is the natural major pathway used to generate acetyl-CoA in this setting to link glycolysis, acetyl-CoA, and histone acetylation in stem cell differentiation (4).

In the facilitation of pluripotency reprogramming by Glis1, a time-dependent correlation is observed between the increase in Glis1 expression and the alteration of acetylated and lactylated histones (87). ChIP-seq indicated the enrichment of pan-Kla and H3K18 la in the promoters at the Oct4, Sall4 and Myc loci, which exhibited patterns parallel to those of H3K27Ac, coordinating with histone acetylation and lactylation in glycolysis-dependent processes such as pluripotency acquisition.

A recent study in CD4^+^ Th1 cells demonstrated that glycolysis is important for acetyl-CoA levels during T cell differentiation (91). Mechanistically, LDHA activity is important for the production of acetyl-CoA, which then enhances H3K9Ac and H3K27Ac levels and IFN-γ expression in Th1 cells (91). In fact, the levels of acetyl-CoA and lactate are altered simultaneously in many cells that depend on glycolysis, and cancer cells and PSCs are common examples.

### Differentiation Decisions

In the M1 macrophage polarization model, ChIP-seq experiments with anti-H3K18la and anti-H3K18Ac antibodies revealed that both of these PTMs are enriched at multiple genes and that H3K18Kla is enriched at specific genes. In particular, increased histone lactylation was found in the promoters of M2-like homeostatic genes, such as Arg1 (79). Notably, histone lactylation was found to exhibit temporal dynamics different from those of histone acetylation in this M1 macrophage polarization system, strengthening the evidence that histone lactylation is functionally distinct from histone acetylation.

### Role of p300 in Lactylation

A triad of transcription factors (HIF-1α, c-MYC and p53) is largely responsible for the glycolytic phenotype in cancers (92). For instance, p53 inhibits the glycolytic pathway by upregulating the expression of p53-induced glycolysis and apoptosis regulator (TIGAR) (93). A cell-free recombinant chromatin-templated histone modification and transcription assay revealed that histone lactylation can directly activate gene transcription in a p53-dependent p300-mediated manner (79).

Evidence suggests that p300 is not only a histone acetyltransferase (94) but also a promising candidate histone lactyltransferase. In addition, p300 tends to be highly enriched at the promoters of pluripotency genes (Oct4, Sall4, and c-Myc) but not somatic genes such as Setbp1, collagen type I alpha 1 chain (Col1a1), and collagen type V alpha 1 chain (Col5a1) during reprogramming.

One critical question that is currently unanswered is how bulk changes in histone acetylation or lactylation are specifically targeted to defined gene programs. Insight into this topic will expand our understanding of the mechanisms that link lactate and acetyl-CoA fluctuations to specific cellular decisions (90).

## Perspectives

### Readers, Writers, and Erasers

**Figure 2:**
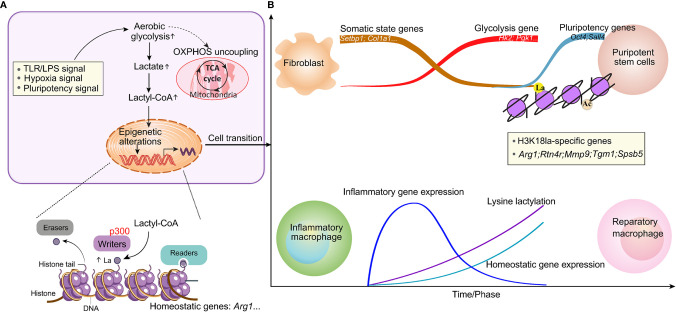
Lactate-induced epigenetic alterations that shape the cellular phenotype. **(A)** Stimuli that activate glycolysis and induce metabolic rewiring can induce epigenetic remodeling through histone modifications, thereby resulting in cellular fate decisions. The enzymes that catalyze the production of the intermediate molecule lactyl-CoA, from which histone lactylation is derived, and those that deposit (writers), remove (erasers) or recognize and interpret (readers) histone lactylation remain to be elucidated. **(B)** An increase in lysine lactylation is delayed after macrophage polarization or somatic cell reprogramming. This delay correlates with changes in the expression of homeostatic genes involved in maintaining biological homeostasis but not with changes in inflammatory gene expression. Lactylation generates a lactate clock to restore normal tissue function after stimulation.

The discovery of lactylation introduces the intriguing possibility of a new window for the Warburg effect. Some possible biochemical links between lactate and lactylation have been proposed, including two metabolic mechanisms, lactyl-CoA is closely related to enzymatic lactylation, and lactyl-glutathione is involved in the acyl-transfer of non-enzymatic lactylation. Further, lactyl-CoA has been detected by liquid chromatography mass spectrometry in mammalian cells and tissues (95). Elucidation of the biochemistry involved in both the transfer of lactyl moieties from lactyl-CoA to histones through p300 or other writer enzymes (79, 84) and the removal of lactyl groups in cellular physiology is an ongoing effort. Similarly, the readers of lysine lactylation that result in cellular changes remain to be identified (**Figure 2**).

Further investigation will clarify more details about how histone lactylation marks are written in a gene-specific manner and how they regulate transcription, as well as about the biochemical process that precisely regulates histone lactation, which may become an important future research topic (69).

### Dynamics of Lactate and Lactylation

Because of the changing microenvironment, the main macrophage phenotype transition is a dynamic process (96). Mouse BMDMs treated with LPS and IFN-γ or with gram-negative bacteria had relatively high H3K18la levels in the promoters of genes that are activated at later time points (24 h) and encode noninflammatory, homeostatic mediators (Arg1), while inflammatory response genes (for example, nitric oxide synthase 2) were induced early (4 h), as expected (79). A study in lung fibrosis showed that the kinetics of Kla induction are slower (24 h) than those of histone acetylation (6 h). This lactylation of histones seemed physiologically relevant, as Kla levels were increased in promoter regions of genes involved mainly in biological processes, such as wound healing. In addition to increasing the expression of Arg1, exogenous lactate also induced increased expression of other anti-inflammatory and proangiogenic genes, including VEGFA, during macrophage activation. Thus, M1 polarization leads to an increase in lactate and the activation of the endogenous ‘lactate clock’, which is generally delayed, and induces the expression of M2-like genes involved in inflammatory repair in macrophages, thus modulating downstream processes regulating homeostatic responses (**Figure 2**).

In this context, understanding the precise relationship between the presence of lactate and histone lactylation, as well as other interconnected metabolite-epigenetic events, on a temporal scale requires further investigation. For instance, the mechanisms controlling the concentration of lactate in microdomains within the nucleus, the lactate concentration required to drive epigenetic reconnection *via* histone lactylation, and the mechanisms by which lactate fluctuation can control a specific gene set in a given tissue are unclear.

The function of lactate and lactylation in various pathophysiological conditions might vary. It will be interesting to explore whether lactate homeostasis can be restored once these conditions have been established.

### Targeted Therapy

Tumors appear to exploit metabolic pathways that are universal and can be beneficial for the resolution of inflammation in a nonmalignant setting, mostly *via* lactate and lactylation. Lactate and its transporters are gaining attention as novel therapeutic targets in such diseases (51). For instance, drugs targeting LDHA, MCT1 and MCT4 are currently being investigated in preclinical studies (97, 98), and the MCT1 inhibitor AZD3956 is being tested in a clinical trial (NCT01791595).

Notably, highly glycolytic tumor metabolism is associated with therapeutic resistance, as observed for the proteasome inhibitor bortezomib in multiple myeloma cells and for carboplatin and paclitaxel in non-small-cell lung cancer cells (99). One explanation is that the acidic TME creates a chemical barrier, resulting in extracellular accumulation of some chemotherapeutic drugs that usually enter cells *via* passive diffusion, which limits their effects and activity (100). Next, given that lactate and acidification foster immune inhibition, the Warburg effect limits the success of immunotherapeutic approaches. Consistent with this observation, the efficacy of adoptive T cell transfer can be limited due to increased tumor glycolytic activity (101). Studies in humans show a correlation between high glycolytic activity in tumors and a low response rate to checkpoint blockade (102).

Overall, targeting lactate metabolism is becoming a useful and promising therapeutic strategy. The discovery of lactylation brings new biological and functional considerations to the historical role of lactate; thus, future research in lactate immunometabolism is expected to provide new drugs that can modulate the activity of immune cells more selectively and with fewer side effects than current drugs (2).

## Conclusions

As highlighted in this review, lactate research has been at the forefront of defining the mechanisms that integrate nutrient signals into metabolite fluctuations and promote epigenetic programming (90). Research on lactylation in macrophages has clarified that the principles underlying these mechanistic events exhibit conservation.

The study of epigenetic regulation of genes by histone lactylation is in its infancy. However, comprehensively defining how lactate metabolism influences epigenetic programming under diverse cellular conditions will be interesting. The mechanistic series of events requiring definition in diverse cellular settings include the mechanisms by which the tumor microenvironment causes fluctuations in metabolites, the effects of metabolite fluctuations on epigenetic modifying complexes and epigenetic events, and the mechanisms by which metabolite-sensitive epigenetic events are translated into specific cellular differentiation gene programs. Evaluation of the regulation of histone lactylation is anticipated to lead to a promising therapeutic approach in cancer.

## Author Contributions

A-NC, YL, Y-HY, J-TF, X-MG, J-PS and JY drafted, edited, and approved the manuscript and figures. All authors contributed to the article and approved the submitted version.

## Funding

This research was supported by the National Natural Science Foundation of China (81772520), Zhejiang Provincial Natural Science Foundation (LGF19H030004), and Zhejiang medical and health technology project (2018PY039).

## Conflict of Interest

The authors declare that the research was conducted in the absence of any commercial or financial relationships that could be construed as a potential conflict of interest.

## Abbreviations

PTM, posttranslational modification; α-KG, α-ketoglutarate; SAM, S-adenosyl methionine; NAD^+^, nicotinamide adenine dinucleotide; IFN-γ, interferon-γ; TCA, tricarboxylic acid cycle; OXPHOS, oxidative phosphorylation; LDH, lactate dehydrogenase; ROS, reactive oxygen species; TME, tumor microenvironment; MCT, monocarboxylic acid transporter; GPR81, G protein-coupled receptor 81; HCA, hydroxycarboxylic acid receptor 1; HIF-1α, hypoxia-inducible factor 1α; VEGF, vascular endothelial growth factor; MDSC, myeloid derived suppressor cell; DC, dendritic cell; CTL, cytotoxic T cell; NK, natural killer cell; NKT, natural killer T; TAM, tumor-associated macrophage; Arg1:arginase 1; JMJ, Jumonji domain-containing; TET, ten-eleven translocation; MSC, mesenchymal stem cell; CAF, cancer-associated fibroblast; IL4, interleukin 4; TGFβ, transforming growth factor β; Kla, lysine lactylation; Kac, lysine acetylation; BMDM, bone marrow derived macrophage; HDAC, histone deacetylase; BCAP, B cell adapter for PI3K; TLR, toll-like receptor; PDGF, platelet-derived growth factor; THBS1, thrombospondin 1; iPSC, induced pluripotent stem cell; Glis1, GGli like transcription factor 1; TIGAR, p53-induced glycolysis and apoptosis regulator; ECM, extracellular matrix; MSC, mesenchymal stem cell.
